# The challenges of implementing a telestroke network: a systematic review and case study

**DOI:** 10.1186/1472-6947-13-125

**Published:** 2013-11-14

**Authors:** Beverley French, Elaine Day, Caroline Watkins, Alison McLoughlin, Jane Fitzgerald, Michael Leathley, Paul Davies, Hedley Emsley, Gary Ford, Damian Jenkinson, Carl May, Mark O’Donnell, Christopher Price, Christopher Sutton, Catherine Lightbody

**Affiliations:** 1 University of Central Lancashire, Preston, UK; 2 Cardiac and Stroke Networks Lancashire and Cumbria, Preston, UK; 3 Lancashire Teaching Hospitals NHS Foundation Trust, Royal Preston Hospital, Preston, UK; 4 North Cumbria University Hospitals NHS Trust, The Cumberland Royal Infirmary, Carlisle, UK; 5 Institute for Ageing and Health, Newcastle University, Royal Victoria Infirmary, Newcastle, UK; 6 NHS Improvement – Stroke, St John’s House, East Street, Leicester, UK; 7 University of Southampton, Faculty of Health Sciences, Southampton, UK; 8 Blackpool Teaching Hospitals NHS Foundation Trust, Blackpool Victoria Hospital, Blackpool, UK; 9 Wansbeck General Hospital, Ashington, Northumberland, UK

## Abstract

**Background:**

The use of telemedicine in acute stroke care can facilitate rapid access to treatment, but the work required to embed any new technology into routine practice is often hidden, and can be challenging. We aimed to collate recommendations and resources to support telestroke implementation.

**Methods:**

Systematic search of healthcare databases and the Internet to identify descriptions of the implementation of telestroke projects; interviews with key stakeholders during the development of one UK telestroke network. Supporting documentation from existing projects was analysed to construct a framework of implementation stages and tasks, and a toolkit of documents. Interviews and literature were analysed with other data sources using Normalisation Process Theory as described in the e-Health Implementation Toolkit.

**Results:**

61 telestroke projects were identified and contacted. Twenty projects provided documents, 13 with published research detailing four stages of telestroke system development, implementation, use, and evaluation. Interviewees identified four main challenges: engaging and maintaining the commitment of a wide range of stakeholders across multiple organisations; addressing clinicians perceptions of evidence, workload, and payback; managing clinical and technical workability across diverse settings; and monitoring how the system is used and reconfigured by users.

**Conclusions:**

Information to guide telestroke implementation is sparse, but available. By using multiple sources of data, sufficient information was collated to construct a web-based toolkit detailing implementation tasks, resources and challenges in the development of a telestroke system for assessment and thrombolysis delivery in acute care. The toolkit is freely available online.

## Background

The thrombolytic drug alteplase is the only widely accepted medical treatment for acute ischaemic stroke. It has been licensed for several years in North America and most European countries, for intravenous use within three hours of stroke, but this restriction, and the need for immediate brain scanning, mean that only a small minority of patients receive the treatment. Although this proportion is increasing, recent estimates range from only 1.4% in UK [1], to 3.7% in USA [2], and 6.6% in Sweden [3].

As well as urgent neuro-imaging, potential candidates for stroke thrombolysis need rapid specialist assessment, which may be required at any time of the day or night. Telemedicine (“telestroke”) has been proposed as a means of providing access to such assessment in hospitals where stroke physicians or neurologists are not always available out of hours [4]. The most common telestroke configuration is a “hub-and-spoke” model, whereby a central “hub” organisation provides remote audio-visual access to a stroke specialist for decision support in a number of peripheral hospitals.

The use of telestroke systems is increasing. An American Heart Association (AHA) survey in 2009 [5] identified a total of 33 organisations providing acute telestroke services (USA 22, Canada 3, Europe & UK 7, Asia 1), compared with 12 identified by a Canadian survey in 2006 [6]. An AHA update in 2012 identified 97 potential telestroke programmes in the USA alone [7]. Systematic reviews of telestroke systems suggest they can improve time to treatment [5,6,8,9], with regional collaborations able to achieve higher rates of thrombolysis than local services working in isolation [10]. Minimum standards for the implementation of telemedicine for stroke care have been published [11], but it is not known how telestroke systems are operationalising this guidance in practice.

The UK Medical Research Council (MRC) framework for complex interventions emphasises the importance of evaluating the process of implementation, as well as clinical and cost effectiveness [12]. This is particularly relevant to telestroke, given the well-known problems of embedding e-health technology initiatives into routine services [13]. Evidence on the characteristics of successful telemedicine applications relates mainly to their use in managing chronic conditions [14,15]. Less is known about systems for emergency care. Recent systematic reviews have pointed to the need for formative methodologies to study telemedicine as a complex and collaborative process [16,17].

As part of a wider project to improve the delivery of thrombolysis in clinical networks, we aimed to develop a web-based “toolkit” to support telestroke implementation, by systematic collation of available guidance for best practice. The guiding framework was Normalisation Process Theory (NPT): an evaluation model that asks what people do to make a complex intervention workable, and to integrate it into practice [18,19]. NPT has been proposed as a suitable framework for evaluating the implementation of complex interventions [20-22], and has previously been used to study the integration of e-health initiatives [13,23-26].

## Methods

The study reported here is part of a larger National Institute for Health Research funded project to support telestroke implementation in one UK region. This paper reports the results of:

• systematic review of the literature to identify good practice recommendations;

• collation and analysis of implementation resources supporting telestroke;

• stakeholder interviews in one UK telestroke project to identify implementation challenges.

### Systematic review

The search targeted telestroke feasibility studies, process evaluations, project descriptions or case studies; qualitative research or surveys of stakeholders; and evaluative or experimental research. We used the short form Cochrane search for stroke, combined with MESH terms and free text words for telemedicine (see Additional file 1). In smaller databases the short search string “stroke and tele*” was used. The following sources were searched:

• databases of published studies: The Cochrane Library, MEDLINE, EMBASE, CINAHL, AMED, PsychInfo, Web of Knowledge, International Bibliography of the Social Sciences (IBSS), Health Management Information Consortium (HMIC) from inception to April 2011;

• *citation searching:* via Web of Knowledge for all included studies;

• *Conference proceedings and Internet searching*: to identify telestroke systems in operation, but not reported in publications.

Two reviewers independently screened records on title and abstract, and filtered all full-text papers for inclusion. The inclusion criteria for papers were:

• *Types of studies:* Publically available studies (2000–2010), containing descriptive, operational, or evaluative data on telestroke systems.

• *Types of participants:* People with suspected or acute stroke, receiving emergency treatment in a health care setting.

• *Types of intervention:* Telestroke systems were defined as the use of audio-visual real-time communication for remote consultation with a decision support provider at a site distant from the patient, for diagnosis or treatment of acute stroke.

Data to be extracted included: description of development, use, or evaluation of a telestroke system; stakeholders’ views on barriers and enablers; reference to “implementation resources” (e.g. standards, policies, specifications, protocols, manuals, memoranda, contracts, decision aids, guidelines, algorithms) to support telestroke system use in acute stroke care; or researcher’s comment on recommendations for best practice (i.e. in discussion). Data extraction and coding were undertaken independently by two reviewers, after training and inter-rater reliability checks. Extracted data were analysed using the Normalisation Process Theory framework, detailing the challenges impacting on telestroke during four stages of system: 1) development, 2) implementation, 3) use, and 4) evaluation. We chose NPT because it is available as a structured analytical framework expressly designed to understand the process of embedding technological systems in health care, and the complexity of the interaction between individuals, new technologies, and context [27]. It comprises 16 dimensions in four categories, illustrated in simplified form from the NPT toolkit [28] in Additional file 2.

### Collation of implementation resources

Identified contacts for telestroke projects were emailed to ask about the public availability of implementation resources referred to in publications or on websites. Content analysis of documents was undertaken to identify the details of discrete implementation tasks.

### Case study of Lancashire and Cumbria telestroke network

We chose to study implementation of a telestroke system in Lancashire and Cumbria (L&C), a geographically diverse area in North West England with long travel times between hospital sites and no obvious central hub organisation. A “network” model, with a roster of specialists from all participating organisations providing out-of-hours clinical decision support, was chosen as the most suitable telestroke service configuration for the area. One organisation was designated lead for governance purposes, and implementation was facilitated by a Cardiac and Stroke Network (CSN): one of 28 regional networks with a remit to support improvement in stroke services in the UK (more information on the features of the telestroke network are provided in Additional file 3). Using this network as a case study facilitated real-time access to people with direct experience of managing telestroke implementation across multiple organisations, rather than relying primarily on the perspective of hub organisations; or on evaluative data from end-users.

Nine purposively sampled stakeholders were interviewed during the year up to July 2011, when the system went live. These included executive, project, and information technology managers from the regional Strategic Health Authority, the CSN, and lead organisation; and doctors and nurses from participating emergency, neurology, and stroke medicine departments. Semi-structured interviews were conducted in the workplace by trained researchers, audio-taped and transcribed. The study was approved by the University of Central Lancashire School of Health Ethics Committee. Approval was requested from the Ethics Committee for the UK Health Service but was not required.

Transcripts were coded using NPT definitions by two trained coders who discussed and agreed final codes. Summaries of key challenges raised were fed back to interviewees (four of whom altered aspects of wording), and were then integrated into an overall summary of challenges within each component of the NPT framework.

## Results

**Figure 1:**
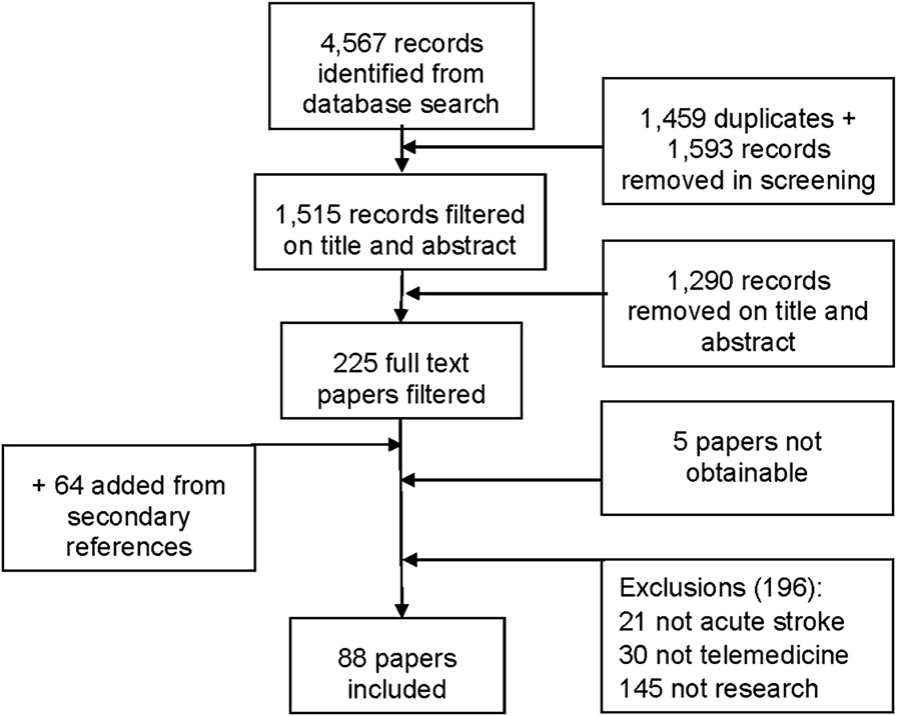
Flowchart of included studies.

The initial search identified 4,567 records, which were screened and filtered as shown in Figure 1.

Internet searching on *Google* (telestroke or stroke + telemedicine) identified 1,290 hits; 218 sites were selected, and 61 telestroke projects identified. Their Internet sites were searched, if available, and principal contacts emailed to ask about publically available implementation resources.

Written information from 20 telestroke projects was located: 13 had published descriptive or evaluative research; and eight projects had resources available on the Internet (Table 1).

**Table 1:** Type of data available from telemedicine projects

**Country**	**Projects**	**Process description, evaluation**	**Outcomes research**	**Internet resources**
**USA**	STROKE-DOC		x	
	REACH, Georgia	x	x	
	TELEBAT, Maryland	x	x	
	Massachusetts		x	
	STARR, Arizona	x		
	SOS, Houston Texas	x	x	
	INTEGRIS, Oklahoma			x
**UK**	East of England			x
	Northumbria			x
	Scottish Telestroke			x
	Avon, Gloucestershire, Wilts,			x
	East Kent			x
**Europe**	Finnish Network	x		
	STENO, Germany		x	
	TEMPiS, Germany	x	x	
	TESS, Germany	x	x	
	Barcelona, Spain	x	x	
**Canada**	Edmonton		x	
	Ontario		x	x
	British Columbia			x
	**Totals**	**8**	**11**	**8**

In addition to project-related papers, we identified a policy statement from the American Heart Association [11,28]; and a national survey of US stroke and emergency physicians’ attitudes to telestroke [29]. Of the eight telestroke projects described, only one (REACH, Georgia) had published a formal process evaluation [30,31]: the remainder were descriptive studies or acceptability surveys included with reports of clinical outcomes, with little methodological detail. Critical appraisal was not attempted.

For each stage of telestroke system 1) development, 2) use, 3) implementation and 4) evaluation, results are presented from the three different data sources in order:

a) research-based recommendations for telestroke implementation;

b) implementation tasks and documentary resources from existing telestroke projects;

c) implementation challenges extracted from case study interviews (interview number in brackets).

### System development

#### ***a) Research-based recommendations***

Development of a telestroke system involves a large number of stakeholders across organisational and professional boundaries. The process can be hampered by lack of alignment with organisational strategy and business processes. Formal collaboration and good communication are needed to negotiate complex inter-organisational issues, and there needs to be a sustainable, long-term business model for investment of financial and other resources, and sharing of expenses and rewards across participating organisations [30,31].

#### ***b) Information from existing projects***

Table 2 describes the kinds of tasks and supporting documentary resources used by existing telestroke projects during system development, either in publications or in Internet resources.

**Table 2:** Tasks and resources needed for system development

***Tasks***	***Example documentary resources***
Develop a case for need, and design a system	Business case
	Service and system specifications
	Site readiness assessments
	Eligibility/resource requirements
Involve key individuals and formalise agreement	Implementation plans/process guides
	Administrator role/responsibilities
	Minutes of meetings/action plans
Formalise financial and organisational agreements.	Letters of co-operation/agreement
	Contracts/accountabilities/liabilities
Facilitate communication and awareness	Insight days
	Bulletins
	Public awareness posters

#### ***c) Information from case study interviews***

In the NPT framework, “coherence” refers to the sense-making work that people need to do individually and collectively about the meaning, use, and utility of a new practice. In the case study, this “sense-making work” involved dealing with the different views of stakeholders on the evidence for the safety, likely costs and benefits of a telestroke system to facilitate thrombolysis.

Uncertainty about the evidence made it difficult to convince managers and commissioners of the need to invest in a telestroke system for the potential benefit of a small minority of stroke patients. The development of a business case was crucial to an appreciation of the potential long-term value of the system. At the time, most published information was based on the hub-and-spoke service model, so there was little guidance on developing a network telestroke model. Although the project Steering Group had a clear vision of the potential benefits of a network model: *“… nothing is disbanded, there is no second class service, everyone is doing thrombolysis,” *(6) they were also aware of the need to involve key individuals to reach agreement about clinical protocols, financial and governance arrangements.

Initially, some clinicians were sceptical about the value of telestroke because of multiple competing priorities, and the perceived difficulties of coordination across organisations and disciplines. Work to develop consensus across diverse staff groups and organisations included employing a Project Manager and a Communications Manager; holding telemedicine insight days and events; providing extensive training opportunities; identifying and working with opinion leaders, and working with individual clinicians to let them experience using the system. Differing views persisted, but the gradual development of consensus around the need for local stroke teams to be involved in thrombolysis delivery meant that any individual reluctance was muted: *“…no one is daring to say they are not keen. Everyone feels it is a “must do”, not for debate, can’t stop it as an individual”* (1).

Communication between organisations was enabled by setting up a Telestroke Executive Group, a Stroke Clinical Advisory Group, and groups of Operational Managers, Continuing Professional Development Leads, Imaging Managers, and Information Technology Leads. Regular meetings of the stroke clinicians were also organised. As well as developing technical and operational specifications, reaching financial agreements was a major challenge. This included negotiating the transfer of responsibilities and costs between different care sectors, all at a time of rapid change in the UK Health Service: *“Implementation dates were far too optimistic, and the lesson learned is that you should get finance written down right from the start. Money has been the biggest hold up”* (3).

### System implementation

#### ***a) Research-based recommendations***

Planners need to assess the compatibility of the telestroke service with existing technical and clinical systems, and agree governance procedures and clinical pathways tailored to local circumstance [30-33].

#### ***b) Information from existing projects***

Table 3 summarises the tasks and documentary resources identified from other projects to support telestroke system implementation.

**Table 3:** Tasks and resources for system implementation

***Tasks***	***Example documentary resources***
Develop shared clinical resources	Pathway analysis
	Process and value stream maps
	Standards/quality criteria for system use
	Joint clinical pathway
Develop governance procedures	Governance policy
	Risk assessment
	Equality & diversity impact assessment
	Privacy impact assessment
	Rotas and job planning
Develop and test the telestroke system	Walkthrough checklist
	Walkthrough action plan
	System maintenance guide
	Troubleshooting guide
	Operational policy

#### ***c) Information from case study interviews***

In the NPT framework, “cognitive participation” refers to the shared work that people need to do to build and sustain a new practice. The main challenge reported during implementation was coordinating shared clinical, technical, and governance procedures across organisations.

Extensive work was undertaken to build the new practice in each organisation, which involved pathway mapping, analysis of training needs, interdisciplinary peer review, and links with existing service improvement initiatives. Because of the network structure, all organisations did feel involved: “*We feel we own the system, we are part of it, we want it to work”* (6). Each organisation had its own key clinicians, who might be neurologists, stroke physicians, or emergency department consultants. Stroke nurse specialists also played a key role in involving other staff in using the system. All participating clinicians had to agree to use standardised policies and recording forms; numerous drafts had to be reviewed within each organisation before final validation: *“Don’t underestimate the time it takes to set up shared pathways, the need for standardised paperwork works fine in theory, but in reality, each site wants to adapt and there is no central system for changing the shared paperwork” *(2).

Even though the network used an externally managed system where all equipment and support was provided by a commercial company, there were difficulties with data compatibility between nine organisations using three different CT imaging systems. The solution was to use an external image hosting site, initially funded by the Regional Health Authority. Equipment maintenance and troubleshooting guides were prepared. Walkthroughs at each site highlighted strengths, weaknesses and gaps in the telestroke process: *“Walkthroughs have been useful, because communication sometimes hasn’t been as good as you thought it was”* (3).

Agreeing a governance structure to clarify where clinical responsibility lay at each stage of the patient pathway was essential. Clinical workloads had to be adjusted, revised consultant job plans agreed, and concerns about unequal shares of workload among organisations addressed. Despite organisational level agreement, individual clinicians and their managers struggled to adapt workloads to cover the additional sessions as well as on-call responsibilities in their own organisations, and issues remained unresolved even as the system went live: *“There are still gaps in the rota, there is still job planning to be sorted out, still areas that have not signed up to it completely*” (8).

### System use

#### ***a) Research-based recommendations***

Potential barriers to telestroke system use in practice include:

• Reluctance because of unfamiliarity; low rates of system use; perceptions of treatment delay; overconfidence in decisions; conflict with cultural norms [34-36]

• Technical problems are fairly rare, but can include problems with sound or image quality; or difficulties/delays getting access to equipment or network [34,36,37]

• Lack of staff confidence or capability in neurological assessment or CT scan reading, fear of clinical complications (e.g. haemorrhage) [32-36]

• Lack of local IT staff to support the technical system [30,31]

• Cultural differences and poor communication routes between disciplines, and centres [30,31].

#### ***b) Information from existing projects***

Table 4 categorises the tasks and documentary resources referred to by existing projects to provide support for telestroke system use.

**Table 4:** Tasks and resources for system use

***Tasks***	***Example documentary resources***
Develop clinical, operational, and technical processes	Prehospital protocol
	Screen/exclude checklist
	CT scan protocol
	Thrombolysis pathway
	Nursing care plan
	Teleconsult recording proforma
	Joint decision record/monitoring
	Contingency plan
	Patient information/consent
	Roles, responsibilities and competencies
Develop staff competency and confidence	Training strategy and needs analysis
	Training priorities, programme objectives
	Training resources
	Competency assessments

#### ***c) Information from case study interviews***

In the NPT framework the term “collective action” refers to the operational work that people do to enact a new practice, involving whether people are able to do what is required of them; whether they have trust in each other; and the necessary skills and resources. Ensuring this operational “workability” required tailoring processes to the unique conditions at each site, while developing and maintaining staff confidence with the technical and cultural changes engendered by the new system.

The siting of telestroke equipment and the need for an internet connection imposed restrictions on the site of clinical care. Decisions about who would provide care depended on the site layout and the availability of skilled staff at different times of day: “*Where are people going to be monitored? and who is going to do it? Accident and Emergency (A&E) staff are used to monitoring, stroke unit staff may not be used to 24 hour continuous monitoring. It needs a Band 5 (registered nurse). If thrombolysis is done in A&E, you are taking a nurse out for an hour. The ideal thing is to get the stroke specialist nurse to take them through the system”* (2). Most centres chose to initiate thrombolysis in the emergency department followed by transfer to an acute stroke or critical care unit for monitoring. Subsequent care was to be provided by the local stroke team, although specialists providing support for thrombolysis decisions usually checked on patients’ progress next day.

Despite extensive training, staff still needed to gain familiarity and confidence with the new system. The need to work closely with people from different organisations meant establishing and adjusting to new working relationships: *“I know how my consultant works, have a pretty good idea the patients he will thrombolyse, the ones he won’t, when he will thrombolyse out of licence. I don’t know whether the other consultants will be a little more cautious when they are talking to a hospital they don’t know with staff they don’t know. You know, they’re not going to know our pathway inside out. They will probably speak to different staff every time”* (7). There was also some concern expressed about whether using the system would affect the quality of the clinical encounter, perhaps reducing the ability to detect subtle clues or to interact meaningfully with the patient and family: *“There may be issues related to preserving privacy and dignity that are less well able to be controlled than in face to face consultation”* (5). Some clinicians were especially worried about using the technology, and trainers commented on the difficulty of maintaining people’s confidence when they might only use the system infrequently.

Wider cultural issues surrounded changes in working patterns and relationships between professional groups, such as involving stroke physicians in reading head CT scans. However, most staff comments concerned the perennial issues of time and work pressures, and whether they could cope with yet another demand, especially one relying on complex technology: *“… in the middle of the night when you’re working with maybe one or two trained staff and 30 patients if you’ve not used it for two months and suddenly you are trying to remember your password at three o’clock in the morning…*” (8).

### System evaluation

#### ***a) Research-based recommendations***

Most published studies on telemedicine system performance have focused on clinical process measures such as: pre-hospital delay; time from admission to initial assessment, specialist consultation, CT scan, blood test results, and initiation of treatment. Other suggestions include monitoring system use, impact on decision making, and acceptability [34-38].

#### ***b) Information from existing projects***

Table 5 details the implementation tasks and quality indicators identified from other published telestroke projects, and the kinds of documentary resources used to support evaluation.

**Table 5:** Tasks and resources for system evaluation

***Tasks***	***Example documentary resources***
Monitor clinical processes and outcomes	Evaluation data flowchart
	Data collection strategy
	Governance reports
Monitor system use and impact on decision making	Decision support log
Monitor fidelity, quality, and acceptability	Patient satisfaction questionnaire
	Staff satisfaction questionnaire

#### ***c) Information from case study interviews***

In the NPT framework, “reflexive monitoring” refers to the appraisal work that people do to assess and understand the ways in which a new set of practices affect them and others around them. Not much information related to evaluation was collected because interviews were mainly conducted during the set-up of the telestroke system, but respondents did comment about how the system was being appraised, used, and reconfigured by users.

Even in the early stages, people commented on the positive impact on patient outcome compared with existing practice: *“I think telemedicine definitely has its place. We’ve had two occasions where (the patient) wouldn’t have been thrombolysed, and they appear to be having a good outcome”* (9). A plan for formal monitoring of care processes and outcomes had been drawn up, but in the first few weeks it was realised that the telestroke system was not only being used for acute stroke diagnosis and treatment, as intended. Clinicians in the A&E department were also using it to seek out-of-hours help for patients with other neurological conditions, so that the demands on the specialist on call were greater than anticipated. Data collection was therefore altered to capture the reasons for telemedicine consultations; and the workload for on-call specialists. This issue was still ongoing when interviews ended.

The interviews also illustrated how attitudes and clinical behaviour throughout the stroke service were influenced by the inevitable comparison between centres, prompted by inter-hospital collaboration, interdisciplinary peer review, and pathway mapping. Staff commented on the ability to learn from each other, and gain new ideas for improving their own service. Participants also mentioned unexpected effects such as raising the profile of stroke care that were unlikely to be captured by formal evaluation: *“It’s really highlighted stroke within our Trust which has been beneficial for the stroke team.”* (7) Others commented on the potential for extended use of the system in the future, such as clinic assessment. However, there was also an appreciation of the possibility of failure of technical innovations: *“it would be awful for it to fail on the first, second or third attempt because if it was to happen then people would just say what a waste of time put a sheet over it and we’ll put in a corner somewhere. That has happened previously in the past with telemedicine video things that have not been used… various things that have just been quietly shoved into a corner because they didn’t work or they weren’t to standard”* (8).

## Discussion

This study involved a search of the main healthcare databases, analysis of documents from existing telestroke projects, and a longitudinal case study of one UK team developing telestroke. The research literature shows limited process evaluation of telestroke, with most of the barriers identified relating to issues of technical and clinical compatibility, and organisational governance. In contrast, findings from the case study tended to highlight the complex relationships between the technical system, and potential users in diverse contexts. The networked nature of the telestroke case study system emphasised the work needed to develop consensus. Factors influencing network workability included the importance of users’ perceptions of evidence, workload, potential, and payback; the amount of “human” work involved in enrolling and maintaining the commitment of a wide range of stakeholders in diverse organisational contexts; and the challenges of managing technical and clinical workability across multiple environments. The facilitating role of the overarching regional Cardiac and Stroke Network was crucial: their actions included pathway mapping, analysis of training needs, providing extensive training opportunities and individualised support, arranging cross-site interdisciplinary peer review and links with existing service improvement initiatives, managing telestroke walkthroughs, and monitoring adjustment of clinical workloads and system use.

Only one other telestroke project has published a process evaluation [30,31]. Our study confirms the importance of harmonising technical, business, and governance systems across organisations but perhaps places more emphasis on the impact of introducing a telestroke system on professional work patterns and clinical care processes, and the amount of time and attention to detail required for implementation work. This is the first process evaluation of a network telestroke model, and it may be that developing horizontal working relationships across multiple organisations needs more intensive preparation than in a hub and spoke configuration, but it is likely that clinical concerns about establishing and adjusting to new working patterns across professional and organisational boundaries are common to all systems. A recent systematic review identified 145 articles relating to the evaluation of telestroke [39] compared with our search in 2009 which yielded 88 research articles, but none of the newer articles are process evaluations, so this type of study is under-represented.

The strengths of our study include combining information from multiple sources (primary, secondary, grey literature sources) to make the complex and often hidden work of implementation more visible. The limitations of this study are the reliance on single case study for primary data collection. The timeframe of the case study was pre-implementation, but this is also the timeframe least studied. We also limited our collation of research literature and documents from existing telestroke systems to those available in the English language, which means we may not have tapped into literature from the strong tradition of telemedicine innovation in European countries. Much process information came from brief descriptions of system commissioning contained within studies focusing on outcome, or unreferenced reports traceable only via the Internet, so little of it could be critically appraised. The most useful information came from excellent implementation support packs from Ontario and Oklahoma, and the materials to support telestroke evaluation from British Columbia. These provided much of the detail for the tasks and resources listed in the tables above.

## Conclusions

Generic toolkits are available to support the implementation of E-Health initiatives [40]. This study affirmed the importance of establishing an implementation process to facilitate mutual adaptation and problem solving across organisational and professional boundaries, and dealing with the clinical challenges that occur when adopting a new technology [15], focusing on the specific example of telestroke. Without published accounts of these formative processes it is not easy to learn from others to proactively design for successful uptake and sustainability of telestroke. To help future projects we have constructed a Standardised Telestroke Toolkit based on the research review, populated with the stroke-specific resources made available by the Lancashire & Cumbria Telestroke Network. It can be accessed at http://www.astute-telestroke.org.uk/[41]. The website also gives detailed information from the L&C stakeholder interviews, so that others can see the amount of work involved and the kind of challenges that occurred, and how they were tackled by the project team. As one stakeholder said: *“I never thought it would take this long – it is a complex piece of work!” *(4).

## Competing interests

The authors declare that there are no competing interests.

## Authors’ contributions

BF Study design, management of systematic review and NPT data analysis, primary author. ED Telestroke network co-ordinator, toolkit advisor, critical reader. CW Principal Investigator, critical reader. AM Synthesis of systematic review, data collection and analysis for case study. JF Data collection and analysis for case study, manuscript preparation. ML Critical review of research design, report and manuscript. PD Critical review of research design, report and manuscript. HE Critical review of research design, report and manuscript. GF Critical review of research design, report and manuscript. DJ Critical review of research design, report and manuscript. CM Critical review of research design, report and manuscript. MOD Critical review of research design, report and manuscript. CP Critical review of research design, report and manuscript. CS Critical review of research design, report and manuscript. CL Trial Coordinator, study design, data analysis for case study, critical review of manuscript. All authors read and approved the final manuscript.

## Pre-publication history

The pre-publication history for this paper can be accessed here:

http://www.biomedcentral.com/1472-6947/13/125/prepub

## Supplementary Material

Additional file 1 MEDLINE search for telestroke. Click here for file

Additional file 2 Four NPT categories and their dimensions. Click here for file

Additional file 3 Details of the Lancashire and Cumbria telestroke network. Click here for file

## References

[B1] Rudd AG Hoffman A Grant R Campbell JT Lowe D Intercollegiate Working Party for Stroke: Stroke thrombolysis in England, Wales and Northern Ireland: how much do we do and how much do we need? J Neurol Neurosurg Psychiatry 2011 82 14 19 10.1136/jnnp.2009.203174 20581132

[B2] Adeoye O Hornung R Khatri P Kleindorfer D Recombinant tissue-type plasminogen activator use for ischemic stroke in the United States: a doubling of treatment rates over the course of 5 years Stroke 2011 42 1952 1955 10.1161/STROKEAHA.110.612358 21636813 PMC4114342

[B3] Eriksson M Jonsson F Appelros P Asberg KH Norrving B Stegmayr B Terent A Asplund K For the Riks-Stroke Collaboration: Dissemination of Thrombolysis for Acute Ischemic Stroke Across a Nation: Experiences From the Swedish Stroke Register, 2003 to 2008 Stroke 2010 41 1115 1122 10.1161/STROKEAHA.109.577106 20395610

[B4] Levine SR Gorman M “Telestroke” : the application of telemedicine for stroke Stroke 1999 30 464 469 10.1161/01.STR.30.2.464 9933289

[B5] Schwamm LH Holloway RG Amarenco P Audebert HJ Bakas T Chumbler NR Handschu R Jauch EC Knight WA Levine SR A review of the evidence for the use of telemedicine within stroke systems of care: a scientific statement from the American Heart Association/American Stroke Association Stroke 2009 40 2616 2634 10.1161/STROKEAHA.109.192360 19423852

[B6] Deshpande A Khoja S McKibbon A Rizo C Jadad AR Telehealth for Acute Stroke Management (Telestroke): Systematic Review and Environmental Scan (Technology overview 37) 2008 Ottawa: Canadian Agency for Drugs and Technologies in Health

[B7] Silva GS Farrell S Shandra E Viswanathan A Schwamm LH The status of telestroke in the United States: a survey of currently active stroke telemedicine programs Stroke 2012 43 2078 2085 10.1161/STROKEAHA.111.645861 22700532

[B8] Demaerschalk BM Miley ML Kiernan TE Bobrow BJ Corday DA Wellik KE Aguilar MI Ingall TJ Dodick DW Brazdys K Stroke telemedicine Mayo Clin Proc 2009 84 53 64 10.4065/84.1.53 19121244 PMC2664571

[B9] Johansson T Wild C Telemedicine in acute stroke management: systematic review Int J Technol Assess Health Care 2010 26 149 155 10.1017/S0266462310000139 20392317

[B10] Price CI Clement F Gray J Donaldson C Ford GA Systematic review of stroke thrombolysis service configuration Expert Rev Neurother 2009 9 211 233 10.1586/14737175.9.2.211 19210196

[B11] Schwamm LH Audebert HJ Amarenco P Chumbler NR Frankel MR George MG Gorelick PB Horton KB Kaste M Lackland DT Recommendations for the implementation of telemedicine within stroke systems of care: a policy statement from the American Heart Association Stroke 2009 40 2635 2660 10.1161/STROKEAHA.109.192361 19423851

[B12] Medical Research Council Developing and Evaluating Complex Interventions: new guidance 2008 London: Medical Research Council

[B13] Mair FS May C Murray E Finch T Understanding the Implementation and Integration of e-Health Services 2009 London: National Institute for Health Research Service Delivery and Organisation Programme

[B14] Broens TH Veld RM H i’t Vollenbroek-Hutten MM Hermens HJ Van Halteren AT Nieuwenhuis LJ Determinants of successful telemedicine implementations: a literature study Journal Telemed Telecare 2007 13 303 309 10.1258/135763307781644951 17785027

[B15] Obstfelder A Engeseth KH Wynn R Characteristics of successfully implementing telemedicine applications Implement Sci 2007 2 25 10.1186/1748-5908-2-25 17662134 PMC1988806

[B16] Ekeland AG Bowes A Flottorp S Effectiveness of telemedicine: a sytematic review of reviews Int J Med Inf 2010 79 736 771 10.1016/j.ijmedinf.2010.08.006 20884286

[B17] Ekeland AG Bowes A Flottorp S Methodologies for assessing telemedicine: a systematic review of reviews Int J Med Inf 2012 81 1 11 10.1016/j.ijmedinf.2011.10.009 22104370

[B18] May CR Finch T Implementing, embedding and integrating practices: an outline of normalization process theory Sociol 2009 43 535 554

[B19] May C Mair F Finch T MacFarlane A Dowrick C Treweek S Rapley T Ballini L Ong B Rogers A Development of a theory of implementation and integration: normalization Process Theory Implement Sci 2009 4 29 10.1186/1748-5908-4-29 19460163 PMC2693517

[B20] May C A rational model for assessing and evaluating complex interventions in health care BMC Health Serv Res 2006 6 86 10.1186/1472-6963-6-86 16827928 PMC1534030

[B21] May C Mair F Dowrick C Finch T Process evaluation for complex interventions in primary care: understanding trials using the normalization process model BMC Fam Pract 2007 8 42 10.1186/1471-2296-8-42 17650326 PMC1950872

[B22] Murray E Treweek S Pope C MacFarlane A Ballini L Dowrick C Finch T Kennedy A Mair F O’Donnell C Normalisation process theory: a framework for developing, evaluating and implementing complex interventions BMC Med 2010 8 63 10.1186/1741-7015-8-63 20961442 PMC2978112

[B23] Boddy D King G Clark JS Heaney D Mair F The influence of context and process when implementing e-health BMC Med Inform Decis Mak 2009 9 9 10.1186/1472-6947-9-9 19183479 PMC2642812

[B24] Elwyn G Legare F Van Der WT Edwards A May C Arduous implementation: does the Normalisation Process Model explain why it’s so difficult to embed decision support technologies for patients in routine clinical practice Implement Sci 2008 3 57 10.1186/1748-5908-3-57 19117509 PMC2631595

[B25] May C Finch T Cornford J Exley C Gately C Kirk S Jenkings K Osbourne J Robinson A Rogers A Integrating telecare for chronic disease management in the community: What needs to be done? BMC Health Serv Res 2011 11 131 10.1186/1472-6963-11-131 21619596 PMC3116473

[B26] Murray E Burns J May C Finch T O’Donnell C Wallace P Mair F Why is it difficult to implement e-health initiatives? A qualitative study Implement Sci 2011 6 6 10.1186/1748-5908-6-6 21244714 PMC3038974

[B27] Finch TL Mair FS O’Donnell C Murray E May CR From theory to 'measurement’ in complex interventions: methodological lessons from the development of an e-health normalisation instrument BMC Med Res Methodol 2012 12 69 10.1186/1471-2288-12-69 22594537 PMC3473304

[B28] May C Finch T Ballini L MacFarlane A Mair F Murray E Treweek S Rapley T Evaluating complex interventions and health technologies using normalization process theory: development of a simplified approach and web-enabled toolkit BMC Health Serv Res 2011 11 245 10.1186/1472-6963-11-245 21961827 PMC3205031

[B29] Moskowitz A Chan YF Bruns J Levine SR Emergency physician and stroke specialist beliefs and expectations regarding telestroke Stroke 2010 41 805 809 10.1161/STROKEAHA.109.574137 20167910 PMC3410649

[B30] Cho S Khasanshina EV Mathiassen L Hess DC Wang S Stachura ME An analysis of business issues in a telestroke project Journal Telemed Telecare 2007 13 257 262 10.1258/135763307781458930 17697514

[B31] Cho S Mathiassen L The role of industry infrastructure in telehealth innovations: a multi-leve analysis of a telestroke program Eur J Inf Syst 2007 16 738 750 10.1057/palgrave.ejis.3000718

[B32] Miley ML Demaerschalk BM Olmstead NL Kiernan TE Corday DA Chikani V Bobrow BJ The state of emergency stroke resources and care in rural Arizona: a platform for telemedicine Telemed J E Health 2009 15 691 699 10.1089/tmj.2009.0018 19694588

[B33] Tatlisumak T Soinila S Kaste M Telestroke networking offers multiple benefits beyond thrombolysis Cerebrovasc Dis 2009 27 21 27 10.1159/000213055 19546538

[B34] Audebert HJ Kukla C Vatankhah B Gotzler B Schenkel J Hofer S Furst A Haberl RL Comparison of tissue plasminogen activator administration management between Telestroke Network hospitals and academic stroke centers: the Telemedical Pilot Project for Integrative Stroke Care in Bavaria/Germany Stroke 2006 37 1822 1827 10.1161/01.STR.0000226741.20629.b2 16763192

[B35] Ickenstein GW Horn M Schenkel J Vatankhah B Bogdahn U Haberl R Audebert HJ The use of telemedicine in combination with a new stroke-code-box significantly increases t-PA use in rural communities Neurocrit Care 2005 3 27 32 10.1385/NCC:3:1:027 16159092

[B36] Wiborg A Widder B Telemedicine in Stroke in Swabia Project: teleneurology to improve stroke care in rural areas: the Telemedicine in Stroke in Swabia (TESS) Project Stroke 2003 34 2951 2956 10.1161/01.STR.0000099125.30731.97 14631092

[B37] Demaerschalk BM Bobrow BJ Raman R Kiernan TE Aguilar MI Ingall TJ Dodick DW Ward MP Richemont PC Brazdys K Stroke team remote evaluation using a digital observation camera in Arizona: the initial mayo clinic experience trial Stroke 2010 41 1251 1258 10.1161/STROKEAHA.109.574509 20431081 PMC2876204

[B38] LaMonte MP Bahouth MN Hu P Pathan MY Yarbrough KL Gunawardane R Crarey P Page W Telemedicine for acute stroke: triumphs and pitfalls Stroke 2003 34 725 728 10.1161/01.STR.0000056945.36583.37 12624298

[B39] Rubin MN Wellik KE Channer DD Demaerschalk BM A systematic review of telestroke Postgrad Med 2013 125 45 50 10.3810/pgm.2013.01.2623 23391670

[B40] Murray E May C Mair F Development and formative evaluation of the e-Health Implementation Toolkit (e-HIT) BMC Med Inform Decis Mak 2010 10 61 9 10.1186/1472-6947-10-61 20955594 PMC2967499

[B41] Standardised Telemedicine Toolkit 4 Strokehttp://www.astute-telestroke.org.uk

