# Supplementary material for: The challenges of implementing a telestroke network: a systematic review and case study
Source: BMC Med Inform Decis Mak. 2013 Nov 14;13:125. doi: 10.1186/1472-6947-13-125 (PMC3833973; doi:10.1186/1472-6947-13-125)
Supplement: Additional file 1 — MEDLINE search for telestroke. [file 1472-6947-13-125-S1.docx]

Additional file 1 MEDLINE SEARCH FOR TELESTROKE

1. cerebrovascular disorders/ or exp basal ganglia cerebrovascular disease/ or exp brain ischemia/ or exp carotid artery diseases/ or cerebrovascular accident/ or exp brain infarction/ or exp cerebrovascular trauma/ or exp hypoxia-ischemia, brain/ or exp intracranial arterial diseases/ or intracranial arteriovenous malformations/ or exp "Intracranial Embolism and Thrombosis"/ or exp intracranial hemorrhages/ or vasospasm, intracranial/ or vertebral artery dissection/

2. (stroke or poststroke or post-stroke or cerebrovasc$ or brain vasc$ or cerebral vasc$ or cva$ or apoplex$ or SAH).tw.

3. ((brain$ or cerebr$ or cerebell$ or intracran$ or intracerebral) adj5 (isch?emi$ or infarct$ or thrombo$ or emboli$ or occlus$)).tw.

4. ((brain$ or cerebr$ or cerebell$ or intracerebral or intracranial or subarachnoid) adj5 (haemorrhage$ or hemorrhage$ or haematoma$ or hematoma$ or bleed$)).tw.

5. hemiplegia/ or exp paresis/

6. (hemipleg$ or hemipar$ or paresis or paretic).tw.

7. 1 or 2 or 3 or 4 or 5 or 6

8. exp Telemedicine/

9. Emergency Medical Service Communication Systems/

10. tele$.tw.

11. 8 or 9 or 10

12. 7 and 11
