# Supplementary material for: The challenges of implementing a telestroke network: a systematic review and case study
Source: BMC Med Inform Decis Mak. 2013 Nov 14;13:125. doi: 10.1186/1472-6947-13-125 (PMC3833973; doi:10.1186/1472-6947-13-125)
Supplement: Additional file 2 — Four NPT categories and their dimensions. [file 1472-6947-13-125-S2.docx]

Additional File 2 Four NPT categories and their dimensions

| **COHERENCE** | **COGNTIVE**  **PARTICIPATION** | **COLLECTIVE ACTION** | **REFLEXIVE**  **MONITORING** |
| --- | --- | --- | --- |
| ***Differentiation***  Can people see how the new practice differs? | ***Initiation***  Who were the key people driving the new practice forward? | ***Interactional workability***  Can people do what the new practice required? | ***Systematisation***  Can people determine the effects of the new practice? |
| ***Communal specification***  Do people agree with the new practice? | ***Enrolment***  Do people agree they should be involved? | ***Relational integration***  Do people feel confident in each other’s work and expertise? | ***Communal appraisal***  Do people agree about the worth of the new practice? |
| ***Individual specification***  Do people understand what they are supposed to do? | ***Legitimation***  Do people organise themselves to undertake the work required? | ***Skill set workability***  Do people have the right skills and training? | ***Individual appraisal***  Do the people involved think the new practice is worth doing? |
| ***Internalisation***  Do people think the new practice has value for them? | ***Activation***  Do people work together to build the procedures required? | ***Contextual integration***  Is the new practice adequately supported and resourced? | ***Reconfiguration***  Do people make changes to the new practice? |

NB “People” can refer to staff or patients
