# Supplementary material for: The challenges of implementing a telestroke network: a systematic review and case study
Source: BMC Med Inform Decis Mak. 2013 Nov 14;13:125. doi: 10.1186/1472-6947-13-125 (PMC3833973; doi:10.1186/1472-6947-13-125)
Supplement: Additional file 3 — Details of the Lancashire and Cumbria telestroke network. [file 1472-6947-13-125-S3.docx]

Additional file 3: Details of the Lancashire and Cumbria Telestroke network

Prior to the implementation of Telestroke, the rate of stroke thrombolysis in Cumbria and Lancashire was estimated to be 0.05%, well below the recommended rate of 10%. Many patients were therefore being denied the opportunity for this effective treatment, simply because of where they lived and the time at which they presented to their local ED. A business plan was developed and the Cumbria and Lancashire Telestroke Network was established to reduce this inequity. The aim of the Network is to deliver expert ‘after hours’ assessment and thrombolysis for patients with acute ischaemic stroke. It serves a wide geographical area in NW England, spanning a distance of 210 km, with a mixed urban and rural population of 2.2 million. The Network is a collaboration between six Acute Trusts across eight hospital sites, and nine Clinical Commissioning Groups. The development and implementation of the Network took two years. As well as the practical aspects of setting up the network and training staff, the development process entailed commissioning, policy development, and agreement of financial and clinical governance arrangements.

The Telestroke rota is covered by 16 consultants. Stroke physicians, general physicians with an interest in stroke, and neurologists from the participating Trusts are eligible to take part in the Network, although there are currently no neurologists on the rota. The on-call consultant utilises teleradiology and videoconferencing to assess the patient and to advise the local team, together with the patient and carer, about appropriate management, including whether thrombolysis treatment would be of benefit. This is normally a one-time process, but the local team may contact the remote consultant for a further consultation if complications arise. After the initial consultation, subsequent patient care is normally handled by the local stroke team and physicians at the admitting Trust. The Network includes one centre which provides neurosurgery and neuro-interventional procedures. Should a patient be deemed to require these services the remote on-call consultant advises the local physicians to refer the patient, and the local team arranges the transfer.

The Network is designed to minimise delays in acute stroke assessment, because it eliminates the need for either patient or consultant to travel to another site. Establishment of a Network, rather than a hub and spoke arrangement, means that a robust stroke infrastructure can be maintained in all Trusts, and stroke unit staff do not become de-skilled in acute stroke care by patients being transferred to a regional stroke centre. This helps to ensure equity in service provision across the Network. Patients and families also benefit from being looked after in their local hospital rather than having to transfer to a distant centre. However, there are concerns that Emergency Department staff might become de-skilled in acute stroke assessment.

Annual costs

Direct costs include technical costs, management support, and on-call costs.

The cost of the ‘Burnbank’ application for teleradiology is shared by all six Trusts within the network and is approximately £1500 (€1766) per site. Overall cost of central management support, including the help desk facility, is £104,000 (€122,459) per year. This is shared *pro rata* between the Trusts, dependent on their stroke activity.

The number of consultants on the on-call rota is also proportionate to stroke activity, with between 1 – 4 consultants per Trust. The on-call rota is negotiated as part of each consultant’s job planning. Some participate in the Telestroke rota instead of undertaking general medical on-call; others get it included within their programmed activities, so consultants do not get additional funding to take the call. The Telestroke rota has greater intensity than a general medical on-call, with more calls after 11pm. Due to practical issues with job planning, it is seldom possible for consultants to be allowed compensatory rest for significant disruption during the previous on-call period.
